# Tri-State Medical Society of Alabama, Georgia and Tennessee

**Published:** 1896-10

**Authors:** 


### Tri-State Medical Society of Alabama, Georgia and Tennessee.

The eighth annual meeting of this highly successful society will be held in Chattanooga, October 13, 14 and 15, 1896.

DOCTOR, you are invited, also to bring your friends. We would be glad to have you to contribute in any way you see proper to the success of the meeting, by reading a paper, discussing cases, or simply by your attendance. Reduced rates on the railroads on the certificate plan. The attendance promises to be large, and the proceedings interesting, as is evidenced by the following partial list of papers:

Convulsions in Children; Report of Cases Treated with Large Doses of Morphine.—Y. L. Abernathy, Hill City, Tenn.

Treatment of Pus in the Pelvis.—W. E. B. Davis, Birmingham, Ala.

Some Remarks on Syphilis.—W. F. Westmoreland, Atlanta.

The Therapy of Antipyretics.—P. L. Brouilette, Huntsville, Ala.

A New Splint for Fractures of the Humerus below the Surgical Neck.—G. A. Baxter, Chattanooga.

Medicine: Hippocratic and Operatic.—Jno. P. Stewart, Attalla, Ala.

The Turkish Bath: Its Therapeutic Indications.—Louise Eleanor Smith, Chattanooga.

Puerperal Eclampsia.—Seale Harris, Union Springs, Ala.

Puerperal Eclampsia.—J. E. George, Rockwood, Tenn.

Humphrey's Operation (Amputation of the Penis), with Presentation of the Patient).—Cooper Holtzclaw, Chattanooga.

................................—Geo. S. Brown, Birmingham.

Diseases and Treatment of the Accessory Sinuses of the Nose. B. F. Travis, Chattanooga.

................................—G. C. Savage, Nashville.

Paper on Eye Diseases.—Alec Sterling, Atlanta.

Paper on Eye Diseases.—J. M. Crawford, Atlanta.

A Statistical Report of Some of the more Recent Remedies Used in the Treatment of Tuberculosis and Summary of Recent Preventative Methods of Value.—R. H. Hayes, Union Springs, Ala.

Operations for Abscess of the Liver.—W. C. Townes, Chattanooga.

Cystitis: Report of Cases.—D. S. Middleton, Rising Fawn, Georgia.

Report of Case of Brachycardia.—W. C. Bilbro, Murfreesboro, Tenn.

Vaginal Hysterectomy for Bilateral Suppurative Processes of the Uterine Adnexa.—W. D. Haggard, Nashville.

The Woodbridge Treatment for Typhoid Fever.—J. W. Duncan, Atlanta.

Diseases of the Veru Montanum (Caput Gallingianis).—W. Frank Glenn, Nashville.

Microscopical and Chemical Examinations as Aids to Diagnosis.—Katherine E. Collins, Atlanta.

Some Obstetrical Complications, with Report of Cases.—R. R. Kime, Atlanta.

Treatment of Skin Cancer.—C. R. Achison, Nashville.

CHATTANOOGA, TENN.

*My Dear Doctor:*

You are invited, with your friends, to attend the Eighth Annual meeting of the Tri-State Medical Society of Alabama, Georgia and Tennessee, to be held in Chattanooga, Tenn., Tuesday, Wednesday and Thursday, October 13, 14 and 15, 1896.

DR. FRANK TRESTER SMITH, Secretary,
Chattanooga, Tenn.

DR. J. B. MURFREE, President,
Nashville, Tenn.

